# Development and Validation of an Interface Between the BIANCA Biophysical Model and Geant4 for Particle Therapy

**DOI:** 10.3390/biomedicines14030542

**Published:** 2026-02-27

**Authors:** Mario P. Carante, Aurora Madonnini, Alice Casali, Ezequiel I. Canay, Ricardo L. Ramos, Francesca Ballarini

**Affiliations:** 1 Physics Department, University of Pavia, Via Bassi 6, I-27100 Pavia, Italy; aurora.madonnini01@universitadipavia.it (A.M.); alice.casali01@universitadipavia.it (A.C.); ecanaycnea@gmail.com (E.I.C.); ricardoluis.ramos@unipv.it (R.L.R.); francesca.ballarini@unipv.it (F.B.); 2 Pavia Section, Istituto Nazionale di Fisica Nucleare, Via Bassi 6, I-27100 Pavia, Italy; 3 National Atomic Energy Commission (CNEA), Buenos Aires C1429BNP, Argentina; 4 Instituto de Tecnología Nuclear Dan Beninson, Universidad Nacional de San Martín (UNSAM), Buenos Aires B1802AYA, Argentina

**Keywords:** biophysical models, Monte Carlo codes, cell survival, particle therapy, fluka, geant4

## Abstract

**Objectives**: The main aim of this study consists of testing the consistency and reliability of the BIANCA (BIophysical ANalysis of Cell death and chromosome Aberrations) biophysical model across different radiation transport codes in the framework of cancer ion-therapy research. **Methods**: Spread-Out Bragg Peak (SOBP) profiles for protons, helium ions and carbon ions were simulated at three different depth ranges (2–3 cm, 5–8 cm, and 10–15 cm) applying two radiation transport codes, FLUKA and Geant4. While BIANCA has been interfaced to FLUKA in a previous work, an interface with Geant4 was purposely developed in this work. Cell survival along all considered SOBP profiles was predicted by BIANCA for two cell lines with very different radiosensitivities: Squamous Cell Carcinoma (SCC), with α/β = 12.68 Gy, and chordoma, with α/β = 2.37 Gy. The agreement between the predictions obtained from the two approaches was quantitatively evaluated by means of Root Mean Square Error (RMSE) and Gamma Index analysis, both for physical dose distributions and for cell survival predictions. **Results**: The comparison between FLUKA and Geant4 simulations demonstrated good agreement. The Gamma Index analysis yielded passing rates exceeding 94.9% for physical dose profiles (criteria: 3%/2 mm) and 96.0% for cell survival probabilities (criteria: 2%/2 mm) across all considered ion species (protons, He, C) and depths. Root Mean Square Error (RMSE) analysis confirmed average discrepancies below 2.5% for physical dose and 1% for biological survival. **Conclusions**: This study shows that the BIANCA model can be applied to predict cell killing along hadron therapy beams when interfaced both with FLUKA and with Geant4. Furthermore, the successful implementation of the interface with Geant4 expands the accessibility and applicability of BIANCA, paving the way for its future integration into different transport codes and/or treatment planning systems.

## 1. Introduction

Hadron therapy has emerged as a very promising modality for cancer treatment, offering improved dose conformity and biological effectiveness compared to conventional radiotherapy [1]. However, the clinical implementation of hadron therapy presents unique challenges that extend beyond physical dose distributions. Especially for ions heavier than protons, the biological effectiveness of hadron therapy beams varies significantly along the beam path due to changes in Linear Energy Transfer (LET) and radiation track structure, particularly in the Bragg peak region and its distal edge [2]. This variation, quantified by means of the Relative Biological Effectiveness (RBE), introduces a layer of complexity to treatment planning that is not present in conventional radiotherapy. While in proton therapy clinical practice a constant RBE value of 1.1 is typically employed, this simplification does not account for the known variations in biological effectiveness that occur along proton SOBPs (e.g., [3,4]). These variations are even more pronounced for heavier ions like helium and carbon [5], for which sophisticated radiobiological models are necessary for accurate treatment planning.

The development of biophysical models capable of predicting cell survival in complex radiation fields has therefore become crucial for optimizing hadron therapy treatments [6]. These models aim to translate physical dose distributions into distributions of Gy-Eq dose (i.e., absorbed dose multiplied by cell survival RBE), enabling more accurate calculation of the dose that has to be delivered both to the tumour and to the surrounding normal tissues. Although at the moment only the Local Effect Model (e.g., [7,8]) and the MKM model (e.g., [9]) are used in clinical practice, several other approaches have been proposed over the years, ranging from empirical to mechanistic models, each with its strengths and limitations. Among these approaches, the BIANCA (BIophysical ANalysis of Cell death and chromosome Aberrations) model has shown promising capabilities for predicting both cell killing and chromosome aberrations (which are related to the risk of secondary tumours) in various irradiation scenarios. 

On the other hand, the accurate delivery of such complex treatments also requires precise modeling of physical interactions, particularly nuclear fragmentation and range uncertainties. In this context, Monte Carlo (MC) simulations are considered the gold standard for validating Treatment Planning Systems (TPSs) and investigating complex mixed-field scenarios [10,11]. Specifically, accurately modeling the fragmentation tail in carbon ion therapy remains a critical challenge for minimizing dose to distal organs at risk [12].

In previous studies, BIANCA has been interfaced with the FLUKA Monte Carlo transport code [13,14], which is used for treatment plan verification at the CNAO centre in Pavia (Italy) and the HIT centre in Heidelberg (Germany). This allowed predicting distributions of cell survival and RBE, as well as chromosome aberrations, in several irradiation scenarios of interest for hadron therapy. In particular, in vitro cell survival has been predicted along SOBP profiles for protons, C-ions and He-ions [15,16]; RBE distributions for side effects in the rat spinal cord have been calculated for C-ion and proton irradiation [17]; distributions of Gy-Eq doses have been (re-)calculated for chordoma patients treated at CNAO with C-ions [18]; and Normal Tissue Complication Probability (NTCP) dose–response curves have been predicted for late tissue reactions [19]. The good agreement of the model predictions with the considered data, as well as with analogous calculations based on the LEM model, suggests that BIANCA can be reliably used for radiobiological modelling in hadron therapy. 

However, relying on a single radiation transport code may represent a limitation for the broader application and validation of the model, also considering that different transport codes employ different physics models, algorithms, and approximations, which can lead to variations in their predictions, particularly in complex radiation fields. FLUKA and Geant4 are among the most widely used codes in medical physics and radiobiology, each with its own strengths, limitations, and user communities. 

Therefore, in the present study, BIANCA was also interfaced to Geant4 [20]. This interface was developed because the GEANT4 user community is also wide, and some users may be interested in hadrontherapy applications. Furthermore, it is useful to have the possibility of interfacing the same biophysical model (in this case, BIANCA) to different radiation transport codes in order to evaluate to what extent the differences related to the physical aspects of the two codes can influence radiobiological outcomes, including cell survival and Gy-Eq dose distributions.

Specifically, distributions of absorbed dose along proton, He-ion and C-ion SOBP profiles at different depths, covering a range of clinical scenarios relevant to hadron therapy, were simulated both with FLUKA and with Geant4. Afterwards, BIANCA was applied to both codes, and distributions of cell survival were predicted for two tumoral cell lines with different radiosensitivities (Squamous Cell Carcinoma (SCC), with a high α/β ratio, and Chordoma, with a low α/β ratio). The comparison between the two approaches, in terms of both absorbed dose and cell survival, allowed for a more comprehensive evaluation of the model performance across different transport codes and different biological systems.

While the BIANCA model has been extensively validated against experimental cell survival data in previous works, the focus of the present study is to verify the consistency of the new Geant4 interface against the reference FLUKA implementation and representative experimental datasets.

## 2. Materials and Methods

The first key step in the validation process consists of a systematic comparison between the predictions obtained with the existing FLUKA-BIANCA interface and those produced by the newly developed Geant4-BIANCA interface. A rigorous benchmarking strategy consists of performing a set of SOBP simulations varying the nature of the incident beam, the location of the plateau, and the irradiated cell line. The chosen approach relies on comparing the cell survival profiles resulting from the same physical dose distributions. This allows for a clear separation between the evaluation of the physical agreement between the two codes—which is expected to be good given the use of similar physics models—and the assessment of radiobiological consistency, which is the primary focus of this validation.

The selected primary particles are protons, helium ions and carbon ions, as they are the most commonly used in clinical hadron therapy. The investigated SOBP positions range from 2 to 3 cm, from 5 to 8 cm, and from 10 to 15 cm This allows for testing the interface across distinct target depths: a very shallow region (typical of ocular tumours), an intermediate one, and a deeper one. The energies of the primary particles used to reproduce these three SOBP positions, for the three different ion types, are reported in Table 1. The weighing algorithm for the monoenergetic beams described in [21,22] was used to produce an almost flat SOBP with FLUKA (FLUKA2025 Version 1.2, INFN (Istituto Nazionale di Fisica Nucleare), Frascati (RM), Italy), used as a reference in this work. After that, the energies and weights of the monochromatic beams were kept fixed for the Geant4 (version 11.4.0, Geant4 Collaboration, Geneva, Switzerland) simulations. Two cell lines are selected for the Geant4-FLUKA comparison, both originating from human tumour tissues. The first one, Squamous Cell Carcinoma (SCC), is relatively radiosensitive, while the second one, Chordoma, is more radioresistant. Their photon radiosensitivity coefficients, α_x_ and β_x_, are 0.379 Gy^−1^ and 0.0299 Gy^−2^, and 0.1567 Gy^−1^ and 0.0661 Gy^−2^, respectively.

By combining the three ion types, three target depths, and two cell lines, a total of eighteen meaningful comparisons between the cell survival depth distributions predicted by the two interfaces can be performed to validate the implementation developed in this work.

The geometry setup consists of an air world volume containing a water phantom shaped as a rectangular box with transverse dimensions of 10 × 10 cm^2^. Cylindrical scoring slices are placed along the beam axis and centered within the water phantom: each slice has a radius of 3 cm and a thickness of 1 mm. The beam source is positioned 1 cm upstream of the phantom entrance and is modelled as a circular, zero-thickness area with a radius of 3 cm. The dose was scored in units of Gy per primary particle, and it was normalized to deliver a uniform physical dose of 2 Gy across the SOBP region.

Concerning Geant4, the physics lists used for the simulations are based on the Hadrontherapy advanced example provided by the Geant4 collaboration [23]. The activated physics models are: G4EmStandardPhysics_option4 and G4EmExtraPhysics for the electromagnetic processes; G4DecayPhysics and G4RadioactiveDecayPhysics for decays; G4HadronElasticPhysicsHP, G4HadronPhysicsQGSP_BIC_HP, G4IonBinaryCascadePhysics, G4StoppingPhysics and G4NeutronTrackingCut for hadronic processes. Only for the case of carbon ions as primary particles, the G4IonBinaryCascadePhysics model was replaced with the G4QuantumMolecularDynamicsModel (QMD), often regarded as more accurate for the fragmentation of nuclei heavier than lithium [24]. Production cuts are set as follows: 10 cm in the world volume, 1 mm in the phantom outside the scoring slices, and 0.1 mm within the slices. Concerning FLUKA, the simulations were performed as described in previous works, like in [7].

In addition to physical dose distributions, cell survival predictions were also performed and compared, using the BIANCA biophysical model, which predicts the induction of cell death and chromosome aberrations for different cells irradiated by different mono-energetic ion beams, from protons to Iron. The model assumes that ionizing radiation induces DNA “Critical Lesions” (CLs), each of which produces two independent chromosome fragments. Fragment un-rejoining, or distance-dependent incorrect rejoining, gives rise to chromosome aberrations, and some aberration types (dicentric chromosomes, rings and large deletions) lead to cell death. The CL yield (mean number of CLs per Gy and per cell) and the chromosome fragment un-rejoining probability are the only two adjustable parameters; a detailed discussion on the model assumptions, as well as a description of the main simulation steps, can be found elsewhere [25]. 

Concerning cell death, V79 cells have been chosen as a reference for BIANCA because they are widely used both in radiobiology research and in the characterization of hadron therapy beams. A large database consisting of linear and quadratic coefficients describing the (simulated) survival of V79 cells irradiated by different mono-energetic ion beams has been obtained by BIANCA ([25] for ions up to Carbon, [15] for ions up to Oxygen, and [26] for ions up to Fe); starting from that database, cell survival can be predicted for any other cell type irradiated with ions up to Fe, provided that the photon survival curve is known. Until now, this has allowed predicting the survival of several cell types, both tumoral (human chordoma, SAOS human osteosarcoma, U2OS human osteosarcoma, human Squamous Cell Carcinoma, Renca mouse tumour) and non-tumoral (Chinese Hamster Ovary cells, normal human skin fibroblasts). In particular, the SCC cell survival database was purposely produced for the present work. The databases of BIANCA are thus completely independent of the radiation transport code and are identical in the case of the interface with both FLUKA and GEANT4.

When interfacing a biophysical model to a radiation transport code for applications to hadron therapy beams, one needs to consider that the cells are exposed to a mixed radiation field. To account for this, dose-averaged linear and quadratic coefficients for cell survival in each irradiated voxel were calculated as follows, according to a formalism developed by Zaider and Rossi [27]:

(1) $$\langle \alpha \rangle =\frac{\sum \alpha_{i}D_{i}}{\sum D_{i}},\;\langle \beta \rangle ={\left(\frac{\sum \sqrt{\beta_{i}}D_{i}}{\sum D_{i}}\right)}^{2},$$

where *D_i_* is the absorbed dose by radiation of type “*i*” (that is, a given particle type of given energy) provided by the transport code, whereas *α_i_* and *β_i_* are the cell survival coefficients for radiation of type “*i*” provided by BIANCA.

Each of the 18 simulations performed in this work consists of 5 independent runs with 2 × 10^6^ primary particles each, for a total of 10^7^ events per SOBP configuration. This allows for the estimation of statistical uncertainties. For each run, depth-dependent quantities are computed and averaged across the 5 runs, and the standard deviation of the mean is calculated as:

(2) $$\sigma_{\langle x\rangle }=\frac{1}{\sqrt{n}}\sqrt{\frac{\sum {\left(x_{i}-\langle x\rangle \right)}^{2}}{n-1}},$$

where *x*_*i*_ is the value of *D*, *α*, or *β* in a given slice from run *i*, ⟨*x*⟩ is the mean value of the same quantity in the same slice, and *n* = 5 is the number of runs. The uncertainty associated with cell survival in a given slice is computed with the usual formulae of error propagation [28]:

(3) $$\sigma_{S}=S\times \sqrt{D^{2}\sigma_{\alpha }^{2}+D^{4}\sigma_{\beta }^{2}+{\left(\alpha +2\beta D\right)}^{2}\sigma_{D}^{2}}\;.$$

To quantitatively evaluate the agreement between the depth–dose profiles and the survival curves simulated with Geant4 and FLUKA, two distinct statistical metrics were employed: the Root Mean Square Error (RMSE) and the Gamma Index analysis. The RMSE provides a global measure of the average discrepancy between the two codes and is defined as:

(4) $$RMSE=\sqrt{\frac{1}{N}\sum_{i=1}^{N}{\left(y_{FLUKA,i}-y_{GEANT4,i}\right)}^{2}}$$

where *y* represents the quantity of interest (normalized physical dose or cell surviving fraction) and *N* is the number of data points. In addition, the Gamma Index (γ) analysis [29] was performed to simultaneously account for dose/effect differences (DD) and distance-to-agreement (DTA), which is particularly relevant in regions with steep gradients such as the Bragg peak or the distal fall-off.

For physical dose comparisons, we adopted the standard clinical acceptance criteria of 3% DD and 2 mm DTA, with a dose threshold of 10%. For the biological endpoints (cell survival), a stricter criterion of 2% DD and 2 mm DTA was applied to rigorously test the biophysical model implementation and to assess possible biological consequences. A global normalization to the maximum value was used, and the agreement was considered acceptable if the passing rate (γ ≤ 1) exceeded 90% and good if it exceeded 95%, in accordance with AAPM TG-218 recommendations [30]. All statistical analyses were performed using custom Python scripts (Python 3.12.3, Python Software Foundation (PSF), Wilmington, Delaware (DE), USA), utilizing the NumPy [31], SciPy [32] and Pandas [33] libraries, while the Gamma Index was calculated using the PyMedPhys library [34].

Finally, the Geant4-BIANCA interface was also benchmarked against experimental data from the literature, on cell survival at different depths along different SOPBs of protons, helium and carbon ions, for cell lines not included in the comparison validation against the FLUKA-BIANCA interface.

## 3. Results

**Figure 1:**
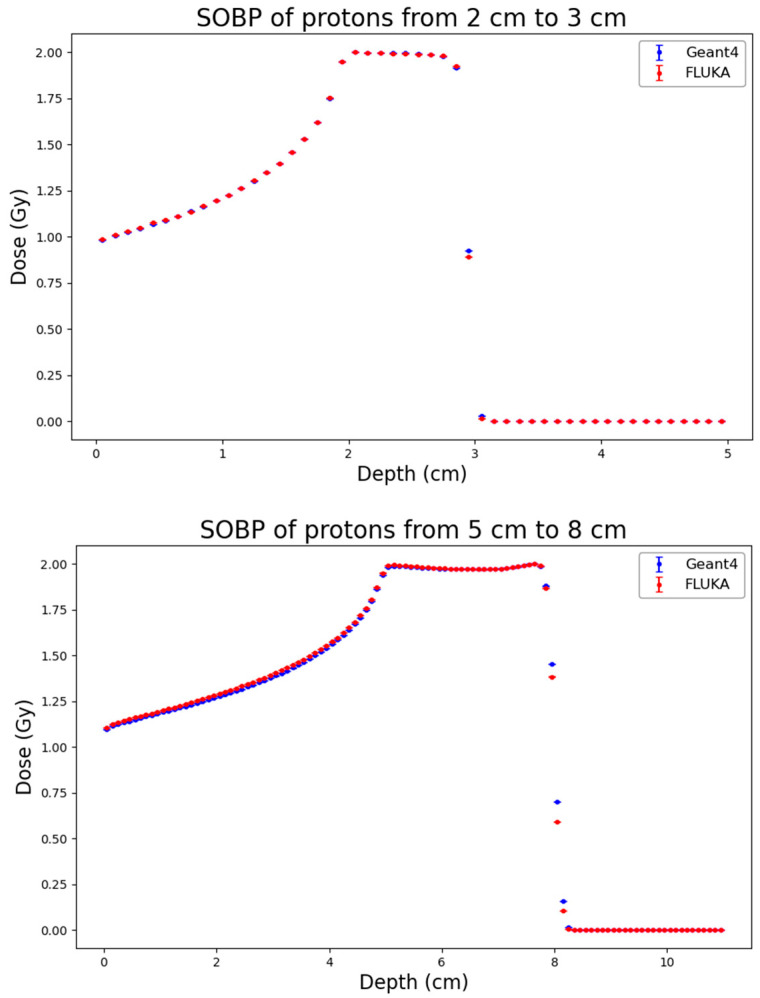

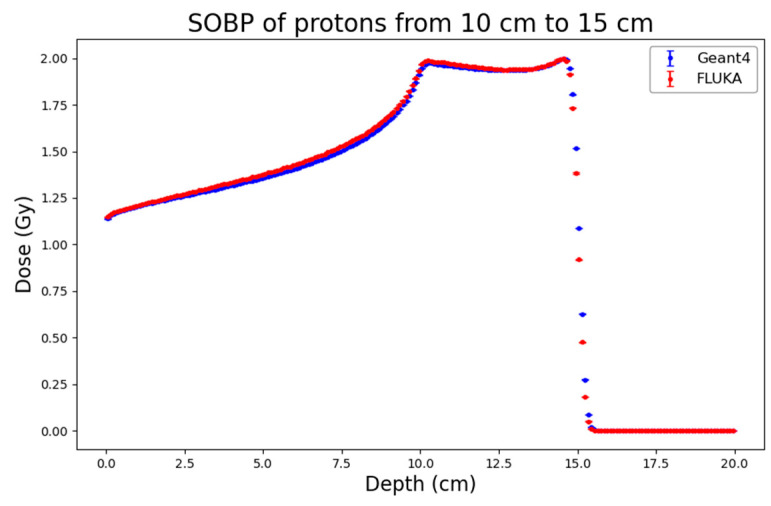
Absorbed-dose distribution for the three considered proton SOBP profiles (from top to bottom: 2–3 cm, 5–8 cm, 10–15 cm) obtained from Geant4 (blue) and FLUKA (red). Statistical uncertainties calculated according to Equation (2) are also reported.

**Figure 2:**
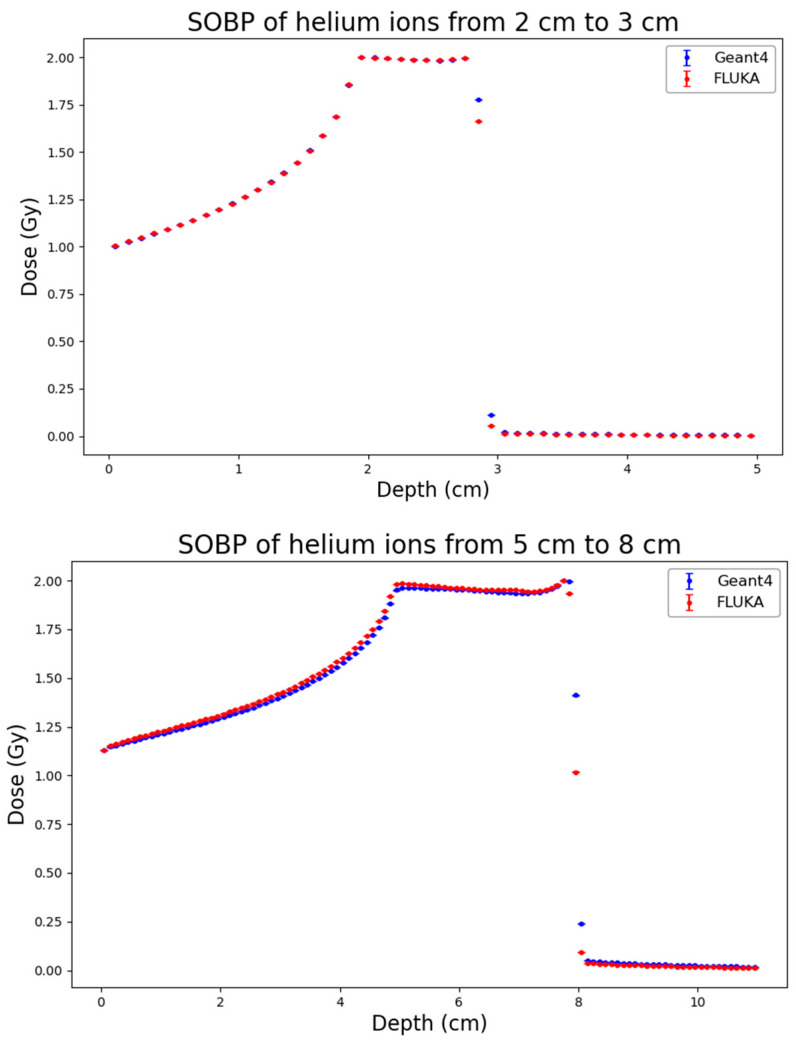

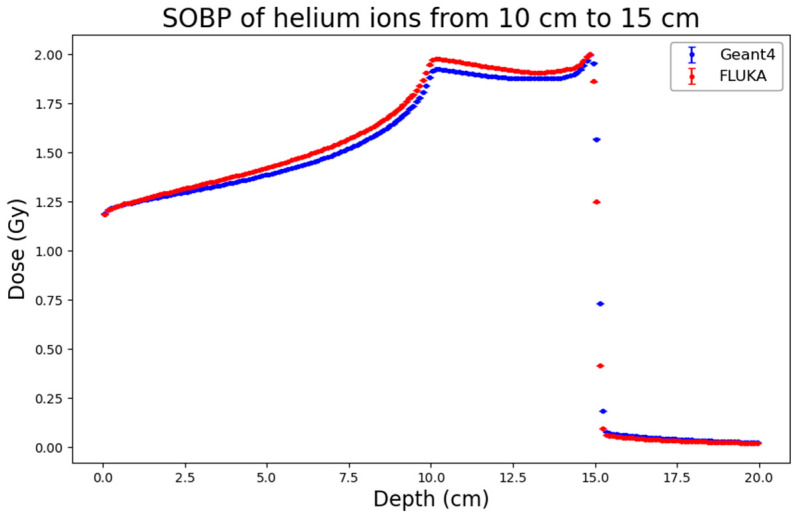
Absorbed-dose distribution for the three considered He-ion SOBP profiles (from top to bottom: 2–3 cm, 5–8 cm, 10–15 cm) obtained from Geant4 (blue) and FLUKA (red). Statistical uncertainties calculated according to Equation (2) are also reported.

**Figure 3:**
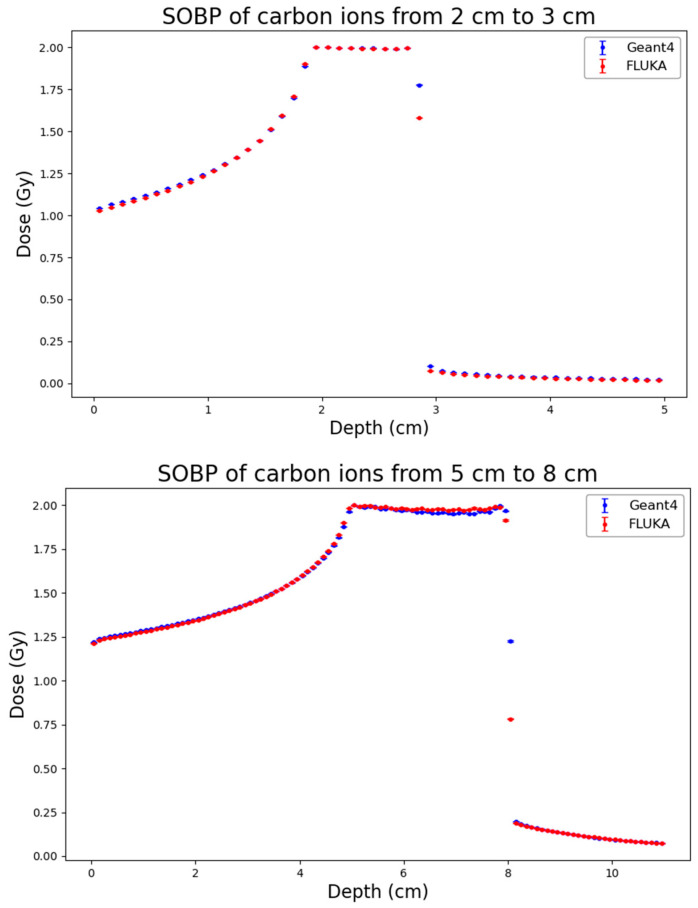

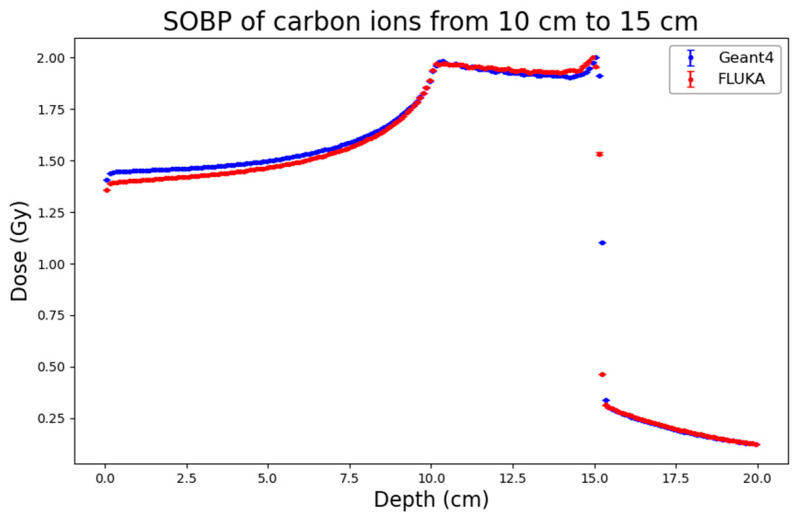
Absorbed-dose distribution for the three considered C-ion SOBP profiles (from top to bottom: 2–3 cm, 5–8 cm, 10–15 cm) obtained from Geant4 (blue) and FLUKA (red). Statistical uncertainties calculated according to Equation (2) are also reported.

As a first step of the work, distributions of physical dose generated by FLUKA and by Geant4 were obtained for the three considered ion types and SOBP profiles. The results, which are reported in Figure 1, Figure 2 and Figure 3, showed high consistency between the two codes across all considered ion species (protons, helium ions and carbon ions) and SOBP depths (2–3 cm, 5–8 cm, and 10–15 cm).

To quantitatively assess the agreement, the RMSE and the Gamma Index (using 3% DD/2 mm DTA criteria) were calculated. The results are summarized in Table 2. For proton beams, the agreement is excellent across all depths, with RMSE values remaining consistently low (ranging from 0.0057 to 0.0248) and Gamma Index passing rates exceeding 97.4% in all cases, reaching 100% for the shallow SOBP (2–3 cm). The mean Gamma values are well below 0.3, confirming that the two codes provide almost identical dose distributions. Helium ions also exhibit a strong agreement, with passing rates consistently above 96.6%. A slight increase in the RMSE and mean Gamma value (0.409) is observed at the largest depth (10–15 cm), likely due to minor differences in the distal region, yet the passing rate remains very high (98.7%). Carbon ions show a similar trend: the agreement is perfect at shallow depths (100% passing rate at 2–3 cm) and remains robust at intermediate depths (98.8%). At the largest depth (10–15 cm), the RMSE increases to 0.0586 and the passing rate decreases to 94.9%. This minor degradation, characterized by a mean Gamma of 0.517, is attributable to the more complex nuclear fragmentation of carbon ions at high energies and larger depths, where differences in the internal physics models of FLUKA and Geant4 become more relevant. However, even in this most challenging scenario, the result is well above the acceptance threshold of 90%.

**Figure 4:**
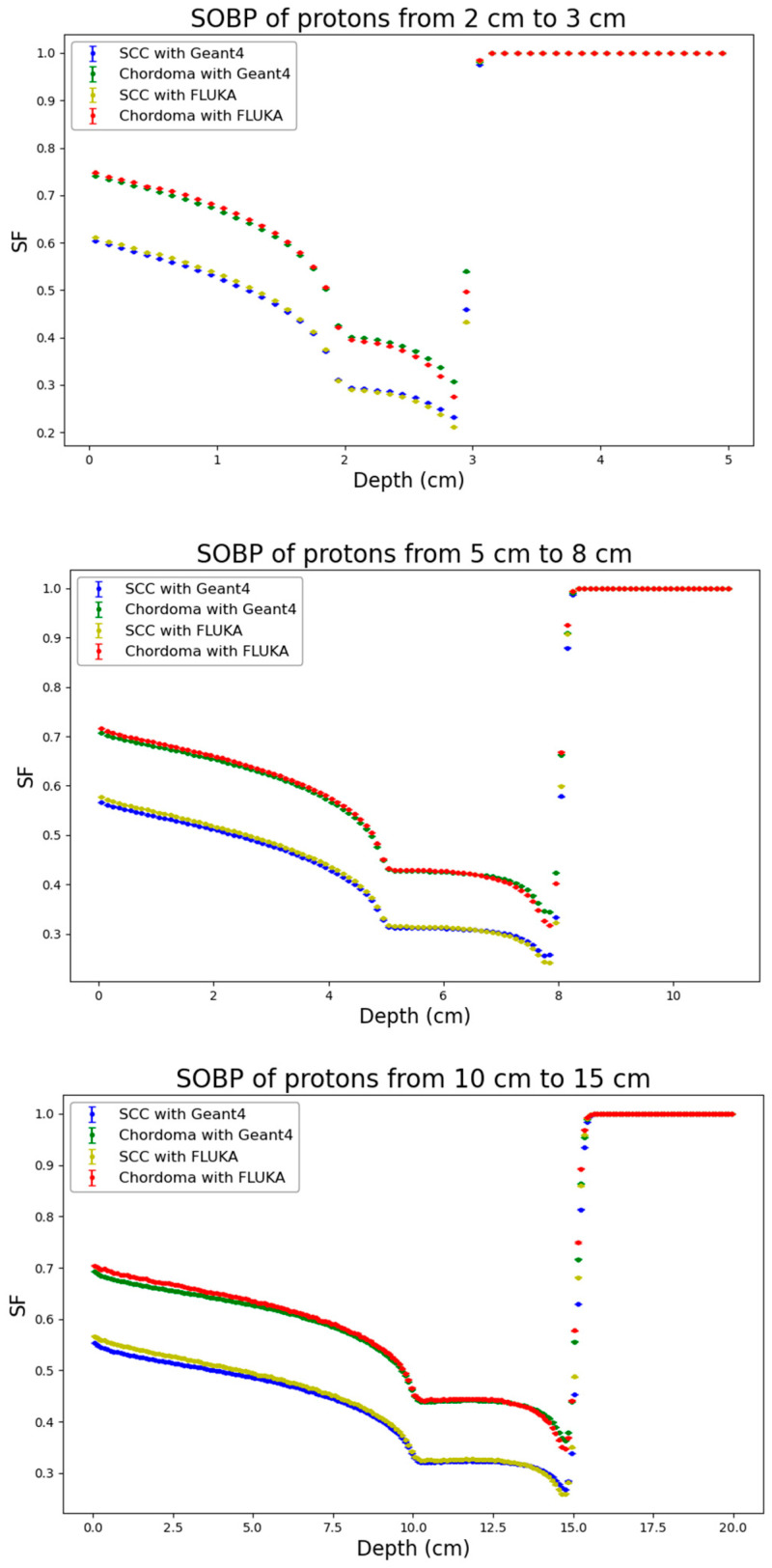
Distribution of the cell surviving fraction (SF) for the three considered proton profiles (from top to bottom: 2–3 cm, 5–8 cm, 10–15 cm) obtained from BIANCA-Geant4 or BIANCA-FLUKA for the two considered cell lines (SCC and Chordoma cells). Statistical uncertainties calculated according to Equation (3) are also reported.

**Figure 5:**
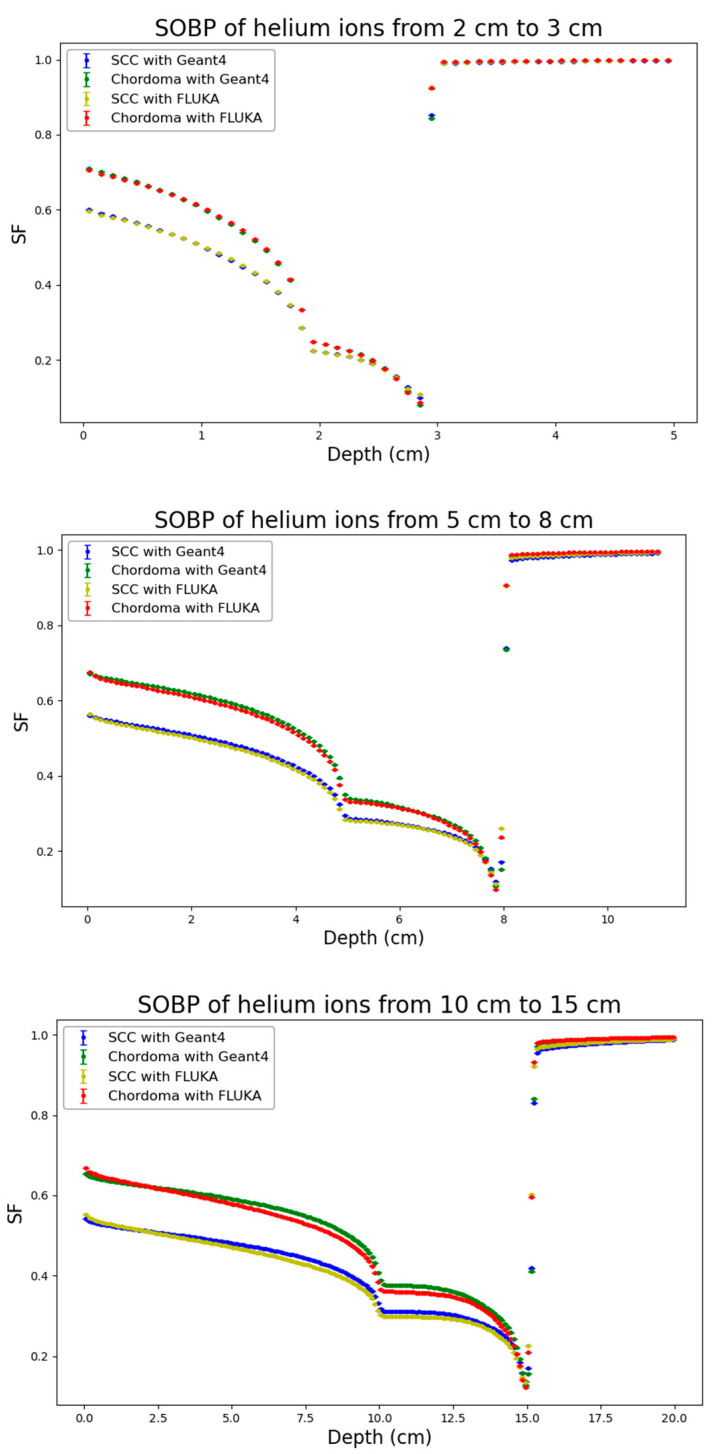
Distribution of the cell surviving fraction (SF) for the three considered He-ion profiles (from top to bottom: 2–3 cm, 5–8 cm, 10–15 cm) obtained from BIANCA-Geant4 or BIANCA-FLUKA for the two considered cell lines (SCC and Chordoma cells). Statistical uncertainties calculated according to Equation (3) are also reported.

**Figure 6:**
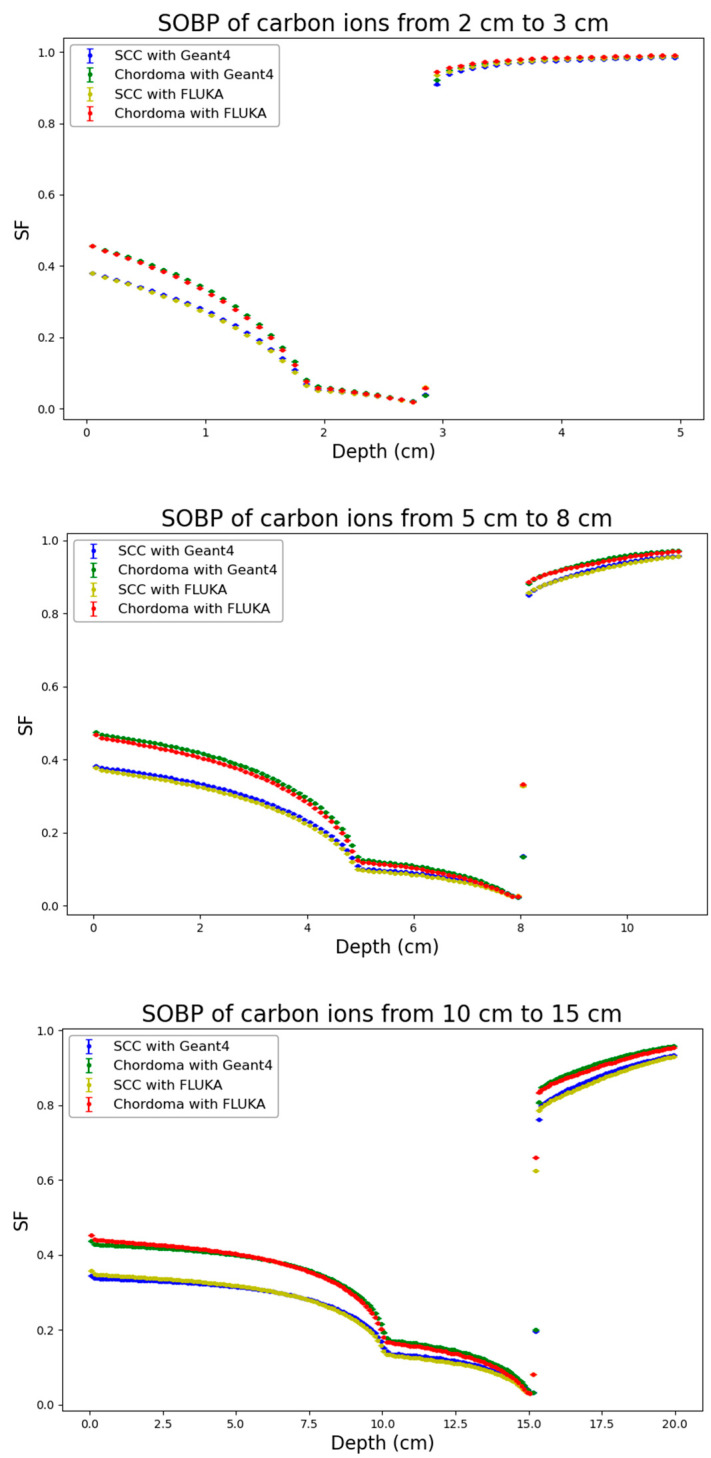
Distribution of the cell surviving fraction (SF) for the three considered C-ion profiles (from top to bottom: 2–3 cm, 5–8 cm, 10–15 cm) obtained from BIANCA-Geant4 or BIANCA-FLUKA for the two considered cell lines (SCC and Chordoma cells). Statistical uncertainties calculated according to Equation (3) are also reported.

Following the physical dose distributions, corresponding distributions of cell surviving fraction generated by the BIANCA-FLUKA or the BIANCA-Geant4 interface as described in the methods were obtained for both considered cell lines (SCC and Chordoma cells). The results are reported in Figure 4, Figure 5 and Figure 6.

Remarkable agreement was found between the predictions obtained by the BIANCA-FLUKA interface and those obtained by interfacing BIANCA with Geant4 for both cell lines, across all considered ion species and SOBP depths. This consistency is particularly noteworthy given the different radiosensitivities of the two cell lines, as reflected by their α/β ratios (12.68 Gy for SCC cells and 2.37 Gy for chordoma cells).

Analogous to the physical dose distributions, the cell survival distributions were also analyzed by applying the RMSE and the Gamma Index analysis, as shown in Table 3. For the biological endpoint, stricter Gamma Index criteria were applied (2% DD/2 mm DTA) to stress-test the biophysical model implementation. Remarkably, the agreement for cell survival was found to be even more robust than for physical dose in several cases. For protons, the Gamma passing rates for both SCC and Chordoma cell lines were consistently high, ranging from 96.0% to 99.1%, with RMSE values below 0.01. For helium ions, the agreement is excellent, with passing rates never dropping below 97.8% and mean Gamma values maintained between 0.15 and 0.30. For carbon ions, despite the physical dose discrepancies observed at 10–15 cm, the biological predictions showed high consistency, with passing rates exceeding 98.6% for all depths and cell lines. For instance, at 5–8 cm, the passing rate was near 99% for both cell lines.

The fact that the passing rates for cell survival (under stricter 2% criteria) are generally comparable to or higher than those for physical dose (under 3% criteria) suggests that the BIANCA model integration in Geant4 is numerically stable. The biological response, which is non-linear, does not amplify the minor physical uncertainties; on the contrary, the biophysical predictions remain robust across different transport codes and biological systems.

The cell survival predictions allow characterizing the decrease in cell survival, and thus the increase in cell killing, along the various SOBP profiles. While this is taken into account in C-ion and He-ion treatment planning by adopting physical-dose distributions that decrease with increasing depth, it is still a matter of debate whether this should be applied in proton therapy, where a constant RBE of 1.1 is generally applied in clinical practice [35]. However, many radiobiological studies show that low-energy protons such as those present in the SOBP distal region have a higher LET, and thus a higher Relative Biological Effectiveness (RBE) with respect to intermediate- and high-energy protons, which explains why the Gy-Eq dose (i.e., the absorbed dose in Gy multiplied by the RBE, which ideally should be constant along the whole SOBP) tends to increase with depth along the SOBP.

**Figure 7:**
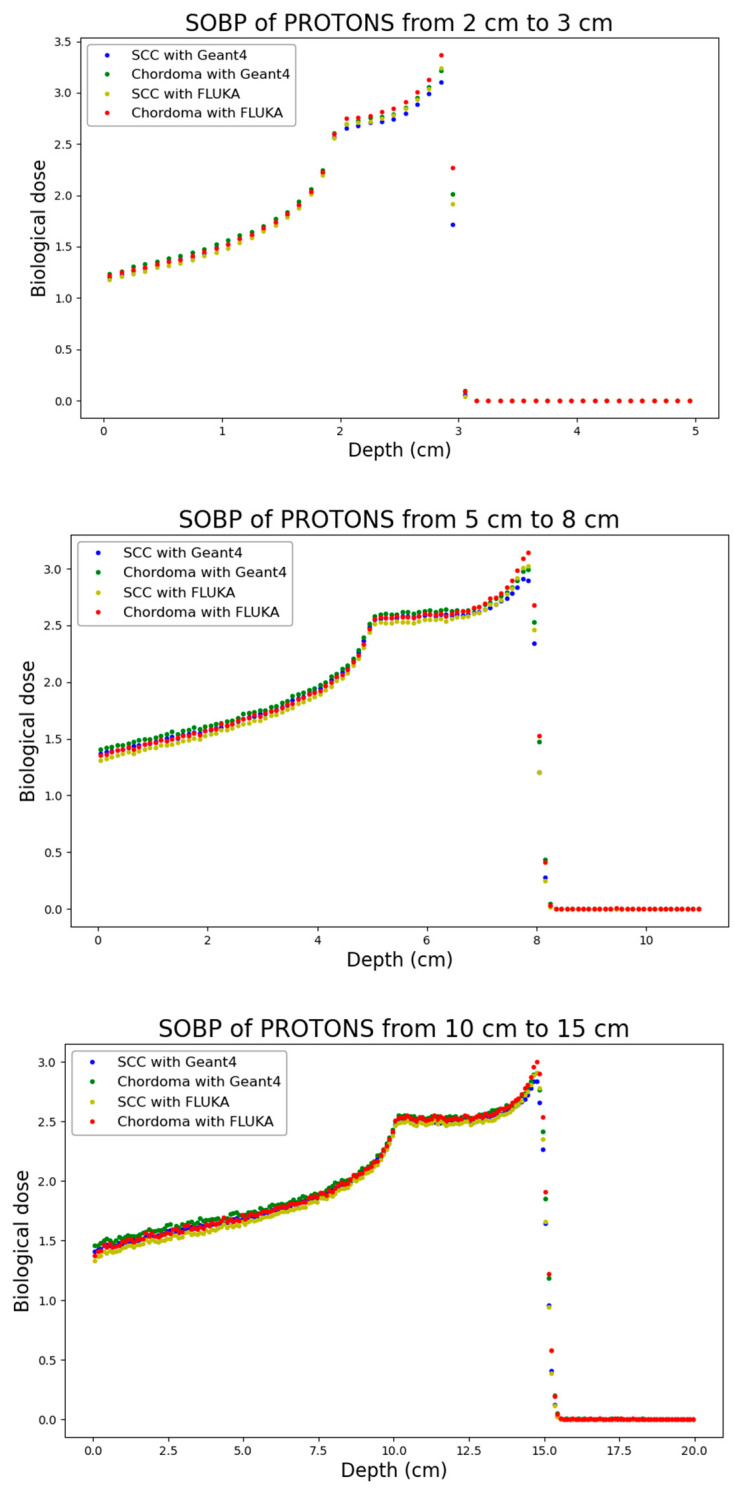
Gy-Eq dose as a function of depth for the three considered proton profiles (from top to bottom: 2–3 cm; 5–8 cm; 10–15 cm).

In this framework, Figure 7 reports the calculated Gy-Eq dose as a function of depth for the three considered proton SOBPs.

**Figure 8:**
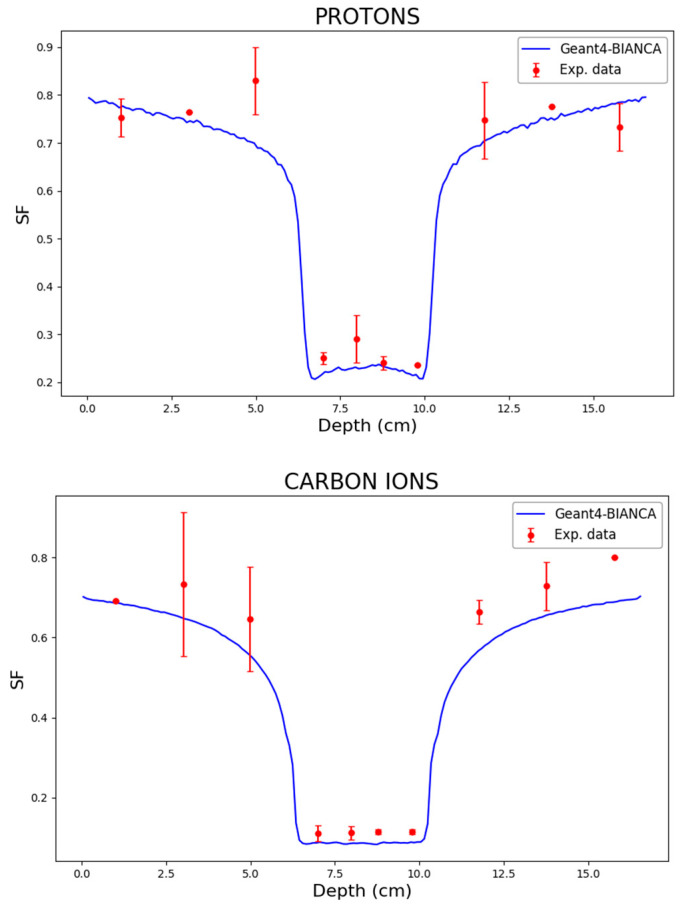
Surviving fraction (SF) as a function of depth for CHO cells irradiated at HIT by two opposing beams of protons (**top**) or C-ions (**bottom**). The lines are predictions obtained in this work by the BIANCA-Geant4 interface, whereas the points are experimental data taken from [2].

After analyzing the aforementioned distributions of physical dose and biological effect in controlled scenarios, where the target was irradiated from a single direction, we tested the BIANCA-Geant4 interface for a scenario that is very frequent in clinical practice, consisting of the target being irradiated by two opposing beams. The simulations, which were performed both for protons and for C ions, were compared with CHO (Chinese Hamster Ovary) cell experimental data obtained at the Heidelberg Ion Therapy (HIT) centre [2], which have been used in a previous work to benchmark the BIANCA-FLUKA interface [15]. In the experimental study, the cells were irradiated by two opposing beams with energy in the range of 90–120 MeV/u (protons) or 175–230 MeV/u (carbon), delivering 1.5 Gy in the entrance channel (for both protons and carbon) and 5.3 Gy (protons) or 3.9 Gy (carbon) in the target center. The irradiations were planned to achieve a homogeneous cell survival level in the target, simulating a 4 cm target located between 6 and 10 cm water-equivalent depth; further details can be found in [2]. The results obtained in this work by the BIANCA-Geant4 interface are reported in Figure 8, which shows good agreement between simulations and data both for protons and for Carbon ions. This suggests that the BIANCA-Geant4 interface developed in this work can be reliably applied not only for single-beam irradiations, but also for two-port irradiations.

**Figure 9:**
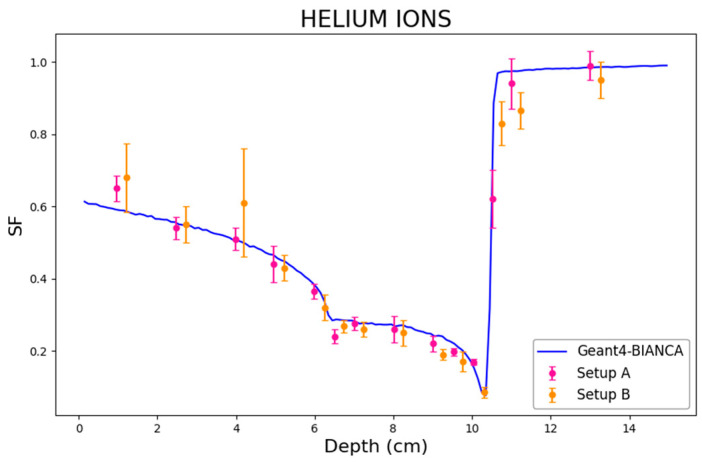
Surviving fraction (SF) as a function of depth for CHO cells irradiated at HIT by He ions. The line represents the predictions obtained in this work by the BIANCA-Geant4 interface, whereas the points are experimental data taken from [36].

In addition to the proton and C-ion experiments described above, CHO cells were also irradiated at HIT by He-ions [36]. Specifically, the cells were irradiated at many different depths along the beam centering the target volume at 8.2 cm depth with a depth range of 4 cm, and the irradiation was planned to obtain a uniform absorbed dose over this region. The physical dose was about 2.4 Gy in the entrance channel and 4 Gy in the SOBP. To increase the resolution in depth and to obtain a denser measurement grid, the cells were irradiated in two configurations (“setup A” and “setup B”), differing from each other only by a small shift in the cell positions along the beam. Further information can be found in the original paper [36]. While the predictions by the BIANCA-FLUKA interface have been reported in a previous work [16], the predictions performed in the present work by the BIANCA-Geant4 interface are reported in Figure 9, which shows very good agreement between simulations and experimental data.

## 4. Discussion and Conclusions

In this work, the BIANCA biophysical model was interfaced with the Geant4 transport code, and distributions of absorbed dose and cell survival levels along proton, He-ion and C-ion SOBP profiles were calculated and compared with analogous distributions obtained by interfacing BIANCA with FLUKA. In all the plots, the error bars due to simulation uncertainties are reported, but they are often so small to be comparable to the dimensions of the points. The good agreement between the two approaches showed the consistency and reliability of the BIANCA predictions across different simulation platforms. Furthermore, distributions of cell survival obtained by the BIANCA-Geant4 interface were compared with experimental data obtained at HIT by irradiating CHO cells by two opposing beams of protons or C-ions, as well as a single He-ion beam.

The results of this study show the robustness and consistency of the BIANCA biophysical model when interfaced with different transport codes. The high level of agreement between FLUKA and Geant4 simulations, both in terms of physical dose distributions and cell survival predictions, validates the newly developed Geant4 interface. The minor discrepancies observed in the distal fall-off and fragmentation tail regions, particularly for carbon ions at larger depths (10–15 cm), can be attributed to the fundamental differences in the nuclear interaction models employed by the two codes. FLUKA utilizes its internal PEANUT (PreEquilibrium-Approach-to-NUclear-Thermalization) model coupled with a RQMD-based approach for heavy ions [37], which handles nuclear fragmentation through a specific coalescence and evaporation chain. In contrast, the Geant4 simulations for carbon ions were performed using the Quantum Molecular Dynamics (QMD) model [38,39], which simulates the interaction on a nucleon-by-nucleon basis. While both models are highly accurate, slight variations in the predicted fragment spectra (mass and angular distribution of secondary fragments like boron and beryllium) inevitably lead to small deviations in the accumulated dose beyond the Bragg peak. However, the Gamma Index analysis confirms that these differences remain within clinically acceptable tolerances.

From a clinical perspective, it is important to assess whether the observed statistical discrepancies, particularly in the distal fall-off region at 10–15 cm, could compromise the sparing of distally located Organs at Risk (OARs). Analysis of the profiles reveals that while local dose differences in the steep fall-off gradient can be numerically significant, they correspond to a spatial range shift of less than 1 mm. In a clinical setting, this sub-millimetric shift should be small compared to the standard safety margins applied to the target volume. Therefore, the dose delivered to potential Organs at Risk (OARs) located distally would not be significantly underestimated or overestimated by the Geant4 interface compared to the FLUKA reference.

On the other hand, the higher agreement in biological predictions compared to physical distributions, even where there are minor physical discrepancies, such as in the fragmentation tail, might seem to imply a lack of sensitivity of the model. However, it is important to emphasize that this convergence is a consequence of the radiobiological response characteristics rather than a limitation in precision. Discrepancies in the physical dose are primarily observed in the distal tail, where the absolute dose levels are low. Due to the exponential nature of cell killing, survival probabilities in these regions remain high. Consequently, even noticeable relative variations in the local physical dose or fragment spectrum translate into small absolute deviations in survival fraction. Conversely, in the high-dose target region (SOBP), the physical engines show good agreement, which is correctly reflected by the biophysical interface. Therefore, the reported ‘robustness’ should be interpreted as the interface’s capability to maintain numerical stability and to correctly process the complex mixed field of particle fragments without introducing artificial bias or amplification of physical uncertainties.

In general, the seemingly paradoxical better agreement for cell survival has a mathematical reason. Starting from the LQ model, it is indeed easy to show that this relationship between the relative difference in the dose and the relative difference in the SF holds [40],

(5) $$\left|\frac{∆S}{S}\right|=(\alpha D+\beta D^{2})\left|\frac{∆D}{D}\right|,$$

showing that a multiplicative factor (*αD + βD*^2^) smaller than 1 leads to a biological difference attenuated with respect to the physical one. This situation may happen for low doses but it also depends on the *α* and *β* values, which vary along the SOBP as a function of depth in a non-trivial way.

The successful implementation of the BIANCA model with Geant4 expands its applicability and accessibility to a wider range of medical physicists and researchers in the field of hadron therapy., This flexibility in code interfacing enhances the model’s potential as a robust tool for the independent validation of Treatment Planning Systems (TPSs) and for the investigation of novel biophysical protocols and research workflows.

It is important to acknowledge that this study was designed as a fundamental validation of the Geant4-BIANCA interface under controlled conditions; therefore, some clinically relevant factors were excluded to isolate the comparison between the transport codes. First, simulations were performed in a homogeneous water phantom. This does not account for tissue heterogeneities (e.g., bone–tissue interfaces or lung cavities), which can introduce range degradation effects. Second, the target was assumed to be static. In a clinical scenario involving Pencil Beam Scanning (PBS), organ motion can lead to interplay effects that degrade dose homogeneity, a factor not addressed in this static comparison. Finally, the current validation focused on normoxic conditions. The impact of tumor hypoxia and the Oxygen Enhancement Ratio (OER), which are critical biological factors in hadron therapy effectiveness, were not evaluated in this work.

Future works may focus on further validation of the BIANCA-Geant4 interface with experimental data, as well as exploration of its use in more complex treatment scenarios, including CT-based patient geometries. The expanded capability of the BIANCA model, now compatible with both FLUKA and Geant4, positions it as a valuable tool for advancing our understanding of radiobiological effects and improving treatment planning in particle therapy.

## Figures and Tables

**Table 1:** Minimum and maximum energies of the primary ions used to produce SOBPs at different positions in depth with protons, helium and carbon ions.

Primary Ion	SOBP Position	E_min_ (MeV)	E_max_ (MeV)
p	2–3 cm	46.2	58.2
p	5–8 cm	77.9	101.8
p	10–15 cm	115.5	145.5
He	2–3 cm	182	229
He	5–8 cm	308	404
He	10–15 cm	460	581
C	2–3 cm	998	1270
C	5–8 cm	1730	2290
C	10–15 cm	2610	3330

**Table 2:** Statistical analysis of the physical dose distributions provided by FLUKA and Geant4, based on RMSE and Gamma Index analysis (criteria: 3%/2 mm).

Ion Type	SOBP Position	RMSE	Pass Rate (%)	Mean Gamma
Protons	2–3 cm	0.0057	100.0	0.089
	5–8 cm	0.0161	97.5	0.164
	10–15 cm	0.0248	97.4	0.205
Helium ions	2–3 cm	0.0182	96.6	0.090
	5–8 cm	0.0437	98.8	0.246
	10–15 cm	0.0473	98.7	0.409
Carbon ions	2–3 cm	0.0290	100.0	0.135
	5–8 cm	0.0439	98.8	0.220
	10–15 cm	0.0586	94.9	0.517

**Table 3:** Statistical analysis of the cell survival distributions provided by BIANCA interfaced with FLUKA and Geant4, based on RMSE and Gamma Index analysis (criteria: 2%/2 mm).

Ion Type	SOBP Position	Cell Line	RMSE	Pass Rate (%)	Mean Gamma
Protons	2–3 cm	SCC	0.0071	96.0	0.233
		Chordoma	0.0098	96.0	0.283
	5–8 cm	SCC	0.0069	98.2	0.170
		Chordoma	0.0067	99.1	0.169
	10–15 cm	SCC	0.0094	98.0	0.234
		Chordoma	0.0080	98.5	0.207
Helium ions	2–3 cm	SCC	0.0108	97.8	0.157
		Chordoma	0.0115	97.8	0.167
	5–8 cm	SCC	0.0187	99.1	0.207
		Chordoma	0.0196	99.1	0.221
	10–15 cm	SCC	0.0179	99.0	0.302
		Chordoma	0.0194	99.0	0.302
Carbon ions	2–3 cm	SCC	0.0062	100.0	0.194
		Chordoma	0.0063	100.0	0.214
	5–8 cm	SCC	0.0195	98.7	0.212
		Chordoma	0.0207	98.7	0.233
	10–15 cm	SCC	0.0313	98.6	0.282
		Chordoma	0.0336	98.7	0.316

## Data Availability

The original contributions presented in this study are included in the article. Further inquiries can be directed to the corresponding author.
